# Identifying Behavior Change Techniques for Digital Interventions Addressing Alcohol and Tobacco Co-Use: Findings From a Delphi Consensus Study

**DOI:** 10.2196/88996

**Published:** 2026-07-03

**Authors:** Anuijan Chandran, Scott Veldhuizen, Kamna Mehra, Laurie Zawertailo, Jurgen Rehm, Christian S Hendershot, Peter Selby, Nadia Minian

**Affiliations:** 1 INTREPID Lab Centre for Addiction and Mental Health Toronto, ON Canada; 2 Institute of Medical Science Temerty Faculty of Medicine University of Toronto Toronto, ON Canada; 3 Department of Family and Community Medicine University of Toronto Toronto, ON Canada; 4 Institute for Mental Health Policy Research Centre for Addiction and Mental Health Toronto, ON Canada; 5 Campbell Family Mental Health Research Institute Centre for Addiction and Mental Health Toronto, ON Canada; 6 Department of Pharmacology and Toxicology University of Toronto Toronto, ON Canada; 7 Department of Psychiatry University of Toronto Toronto, ON Canada; 8 Dalla Lana School of Public Health University of Toronto Toronto, ON Canada; 9 Department of Population and Public Health Sciences Keck School of Medicine of the University of Southern California Los Angeles, CA United States; 10 Institute for Addiction Science Keck School of Medicine of the University of Southern California Los Angeles, CA United States; 11 Department of Psychiatry & the Behavioral Sciences Keck School of Medicine of the University of Southern California Los Angeles, CA United States; 12 Department of Psychology University of Southern California Los Angeles, CA United States

**Keywords:** Delphi study, alcohol, tobacco, reduction, cessation, behavior change techniques

## Abstract

**Background:**

Alcohol and tobacco use frequently co-occur and contribute significantly to the global burden of disease. Despite the well-established benefits of addressing both behaviors simultaneously, health care professionals often face substantial challenges in delivering integrated interventions, including limited time, training, and resources. Digital health interventions offer a promising avenue to directly support patients in reducing alcohol and tobacco use, while bypassing some of the barriers encountered in clinical settings. However, there is a lack of consensus on the key behavior change techniques (BCTs) that must be incorporated to ensure that interventions are evidence based and contextually appropriate, making them effective.

**Objective:**

The study aims to identify expert opinions on the most suitable and effective BCTs (reflecting both behavioral relevance and delivery feasibility) to be included in a 1-time, self-guided digital intervention intended to initiate behavior change and support alcohol reduction among people trying to quit smoking.

**Methods:**

We conducted a 2-round modified Delphi study with 14 panelists with expertise in behavioral science, alcohol and tobacco treatment, and digital interventions. Panelists rated 20 BCTs identified in a previous rapid review using the acceptability, practicability, effectiveness, affordability, safety, and equity (APEASE) criteria. BCTs were deemed “appropriate” if at least 70% (n=10) of panelists agreed on all criteria.

**Results:**

Six BCTs were identified as appropriate for implementation: goal setting, action planning (individualized change plan), action planning (reduction strategies), feedback on behavior, reattribution, and pros and cons. These BCTs were considered effective for promoting behavior change through structured planning and personalized strategies. The panel reached partial consensus on several BCTs, while 8 BCTs were deemed inappropriate for a 1-time, unsupervised digital intervention.

**Conclusions:**

The results of this study offer a consensus-based view, reflecting expert opinion on the perceived appropriateness and feasibility of the BCTs that should be included in a 1-time digital intervention to address co-occurring alcohol and tobacco use.

## Introduction

The concurrent use of tobacco and alcohol is a significant public health concern globally [1]. The use of these substances contributes substantially to the burden of chronic diseases, including various cancers, cardiovascular diseases, and respiratory conditions [2-4]. For example, the combined use of alcohol and tobacco accounts for 85% of the population attributable risk (PAR) for hypopharyngeal and laryngeal cancers, with approximately half of this risk attributable specifically to concurrent use [5]. Similarly, a substantial proportion of oropharyngeal (PAR=74%), esophageal (PAR=67%), and oral cancers (PAR=61%) are linked to alcohol and tobacco, with concurrent use contributing 44%, 39%, and 40% of the risk, respectively [5]. Alcohol use has also been shown to decrease the likelihood of successful smoking cessation, thereby indirectly increasing the risk of smoking-related diseases through prolonged tobacco use [6].

Recognizing the synergistic health risks of tobacco and alcohol use, clinical guidelines recommend that these behaviors be addressed together using integrated interventions [7-9], and people who use both substances often express a preference for receiving support that targets both behaviors simultaneously [10]. Despite these recommendations and patient preferences, uptake of integrated approaches in clinical practice remains limited. In Ontario, Canada, few primary care professionals routinely deliver brief alcohol interventions, and tobacco and alcohol treatments are typically offered as separate services [9,11]. Although health care professionals delivering smoking cessation supports acknowledge the importance of screening for alcohol use and delivering an appropriate intervention [12], commonly cited barriers include time constraints, limited training, and concerns about potentially stigmatizing patients [13].

To address these implementation challenges, direct-to-patient digital health solutions, such as patient portals, offer a promising avenue for delivering integrated care. Patient portals can facilitate access to evidence-based interventions, reduce stigma, provide tailored health information, and promote patient engagement [14]. They are increasingly recognized as a cost-effective and equitable platform for implementing interventions targeting co-occurring substance use behaviors [14].

There is growing evidence that digital interventions delivered via portals can reduce alcohol consumption and support adherence to low-risk drinking guidelines [15]. A recent meta-analysis found that such interventions reduced total daily alcohol intake by 4.1 g, equivalent to approximately 0.3 standard drinks per day (95% CI 2.9-5.4), and nonsignificantly decreased the frequency of heavy episodic drinking by 1.11 episodes per month (95% CI 0.32-1.91) [16].

Brief interventions (BIs) in primary care are evidence-based strategies that can reduce risky alcohol use and support smoking cessation [17]. The 2023 Canadian Clinical Guideline for High-Risk Drinking and Alcohol Use Disorder recommends offering a BI to all patients who screen positive for high-risk alcohol use, defined as exceeding Canada’s Low-Risk Alcohol Drinking Guidelines: consuming >2 standard drinks per day on average, consuming >10 drinks per week for women and >15 drinks per week for men, or engaging in heavy episodic drinking (consuming ≥4 drinks for women or ≥5 drinks for men on a single occasion) [18]. Although traditional BIs often involve multiple face-to-face counseling sessions, evidence supports the efficacy of shorter formats [19]. A Cochrane review found that BIs delivered in primary care can reduce alcohol consumption, even when delivered in short sessions [20]. Additionally, electronic screening and BIs (eSBIs), digital adaptations of BIs, have shown effectiveness in reducing risky drinking without placing additional demands on health care professionals [21]. However, it remains unclear which components or behavior change techniques (BCTs) are most appropriate and effective for inclusion in such interventions.

To address this gap, we first conducted a rapid systematic review (PROSPERO: CRD42023445492) to identify expert opinions concerning BCTs with demonstrated effectiveness in reducing both tobacco and alcohol use. BCTs are defined as “observable, replicable, and irreducible components of an intervention designed to alter or redirect causal processes regulating behaviours” [22]. The review identified 16 candidate BCTs associated with reductions in both behaviors: goal setting, action planning, problem solving, information about health consequences, feedback on behavior, information about antecedents, pros and cons, self-monitoring, social support, social comparison, behavior substitution, information about social and environmental consequences, behavioral practice rehearsal, credible source, comparative imagining of future outcomes, and nonspecific reward.

In August 2022, Ontario’s largest smoking cessation initiative—the Smoking Treatment for Ontario Patients (STOP) program—launched the STOP patient portal, a secure, self-guided digital platform designed to support self-enrollment, baseline assessments, and access to tailored resources prior to a clinical appointment. The STOP program, delivered through more than 300 primary care clinics, community health centers, mental health and addiction centers, and other treatment settings, provides free nicotine replacement therapy and behavioral support to a large, diverse population, including individuals with lower income and education levels; those from rural areas; and many living with mental health, physical health, or substance use challenges [23].

As part of the patient portal’s registration process, users complete a mandatory baseline survey that includes the Alcohol Use Disorders Identification Test–Consumption, a validated 3-item screening tool that identifies individuals who engage in hazardous drinking or have alcohol use disorders [24]. This digital infrastructure presents a scalable and accessible opportunity to embed a brief, direct-to-patient alcohol intervention, delivered without requiring clinician involvement, into the existing system. Patient portals are increasingly being used to deliver preventive health interventions, including eSBIs for alcohol use. These platforms enable automated, tailored feedback and can reduce barriers related to time constraints, stigma, and limited clinician capacity. Randomized controlled trials have demonstrated that digital alcohol interventions can reduce weekly consumption and heavy episodic drinking [25]. Additionally, eSBI has been shown to significantly reduce alcohol consumption in nontreatment-seeking populations [26]. Previous research has also demonstrated that web-based alcohol interventions are generally well accepted and show potential for reducing consumption [27].

Despite this growing evidence, there is limited guidance on designing these interventions within patient portals, particularly regarding which BCTs are most appropriate, feasible, and effective for brief, self-guided delivery.

To address this gap, we aimed to establish consensus among experts in digital health, substance use treatment, behavioral science, and implementation science on the most important BCTs for inclusion in a direct-to-patient digital intervention targeting concurrent tobacco and alcohol use. The goal of this study was to identify BCTs considered appropriate within the constraints of the delivery format (ie, a 1-time, self-guided digital intervention), rather than to rank BCTs according to their comparative behavioral effectiveness. Therefore, expert ratings reflect perceived appropriateness and feasibility within this specific implementation context. Given the complexity of this task and the diversity of expertise required, we selected the Delphi method, which is well suited to synthesizing diverse perspectives and generating consensus in areas where empirical evidence is emerging or context-specific decisions are needed [28]. This manuscript describes a Delphi study conducted to identify the most suitable and effective BCTs from a rapid systematic review for inclusion in the STOP patient portal. To ensure consistency with current terminology, we mapped the identified BCTs to their corresponding Behaviour Change Intervention Ontology (BCIO) identifiers [29]. The BCIO was released after the start of our study; therefore, we report both the original BCT labels from the V1 taxonomy tool [30] and the updated BCIO labels. This dual reporting approach ensures transparency, reproducibility, and alignment with the latest standards in behavioral science and digital health research.

## Methods

### Study Design

We conducted 2 rounds of a modified Delphi exercise to establish expert consensus on the most important BCTs for inclusion in a digital intervention targeting co-occurring tobacco and alcohol use. We followed the Conducting and Reporting Delphi Studies (CREDES) guidelines [31] to guide the process.

We selected a modified Delphi methodology to enable structured, iterative input from a panel of experts while allowing asynchronous participation. In this study, we modified the classical Delphi approach [32] by presenting a predefined list of BCTs identified in the rapid review, rather than starting with open-ended questions. This modification streamlined the process, increased efficiency, and allowed participants to focus their expertise on evaluating and refining a curated set of concepts [33].

### Questionnaire Development or Survey Structure

The research team developed a questionnaire based on findings from our rapid systematic review [34], which identified 27 commonly used BCTs in interventions targeting co-occurring alcohol and tobacco use. From these, 16 BCTs were prioritized for expert evaluation based on the research team’s assessment of their relevance and feasibility within the context of a 1-time, direct-to-patient digital intervention delivered through the STOP patient portal. BCTs were excluded if they required clinician involvement, medications, or repeated engagement that could not be accommodated within the digital portal. For example, pharmacological support (BCT 11.1; BCIO:007144) was not included because it typically involves medications that require a prescription, and their use must be carefully assessed by a health care professional. A 1-time digital intervention cannot provide the medical assessment, diagnosis, or prescription needed to determine if pharmacological support is appropriate for an individual, nor can it ensure proper supervision during use. Moreover, the STOP program already provides pharmacological support for smoking cessation through nicotine replacement therapy, which is not a prescription medication.

To facilitate more precise feedback, 3 BCTs—action planning (BCT 1.4; BCIO:007010), problem solving (BCT 1.2; BCIO:007008), and information about antecedents (BCT 4.2; BCIO:007052)—were disaggregated into their core components to better reflect how each could be operationalized within this format. Specifically, “problem solving” was divided into 3 items (identifying barriers, craving management strategies, and recognizing high-risk situations), action planning into 2 items (developing individualized change plans and reduction strategies), and information about antecedents into 2 items (providing general information about antecedents and explaining the link between smoking and drinking). This resulted in 20 items for expert rating. Each BCT was presented with a brief definition and an illustrative example drawn from the reviewed studies to support informed appraisal. Panelists were asked to rate each BCT on the acceptability, practicability, effectiveness, affordability, safety, and equity (APEASE) criteria [35]. The APEASE framework provides a structured approach to evaluating intervention components in terms of their real-world feasibility, impact, and alignment with public health values. Ratings took the form of an ordinal scale, with options ranging from “strongly agree” to “strongly disagree,” as well as an “uncertain” option. Additionally, participants were encouraged to provide free-text comments for each intervention strategy and to suggest additional alcohol intervention strategies that the STOP patient portal should include, allowing reconsideration of any potentially relevant BCTs during the consensus process.

In round 2, participants were invited to rerate BCTs that had not achieved consensus in the first round. They were also shown anonymized summaries of group-level ratings and qualitative feedback from round 1 to facilitate reflection on, and reconsideration of, their initial responses. Both rounds were delivered using Research Electronic Data Capture (REDCap; Vanderbilt University), a secure web-based platform for managing research data.

### Expert Panel: Eligibility and Recruitment

Panelists were eligible to participate if they had expertise in behavior change, tobacco and alcohol treatment, and digital interventions for substance use, as determined by the research team based on academic publications, clinical experience, or relevant program involvement. Eligible individuals included researchers, clinicians, and implementation specialists with relevant academic or professional experience.

We used a combination of convenience and snowball sampling techniques [36] to recruit a diverse panel. Members of the investigator team leveraged their professional networks and distributed a virtual recruitment flyer via email to 34 experts. In addition, personalized invitations were sent to 14 authors of the studies included in the rapid review, given their familiarity with the literature and relevant intervention components. Although there is no universally agreed-upon ideal sample size for Delphi studies, a minimum of 8 panelists is generally considered sufficient to support consensus building [37,38]. To ensure diverse perspectives and account for potential attrition, we aimed to recruit up to 15 panelists. All panelists took part in the Delphi study voluntarily, and their responses were collected anonymously.

### Study Procedures

The first survey was administered between August and September 2024, followed by a second round from November 2024 to January 2025. Panelists who agreed to participate were provided with a briefing note (Multimedia Appendix 1) and a link to the REDCap survey. The briefing note included (1) information about the sociodemographic characteristics of the STOP patient population, (2) details of the clinical treatment participants receive for smoking cessation, and (3) a list of BCTs identified by the rapid review as effective in addressing alcohol and tobacco use.

Once panelists opened the survey, they were directed to a consent form. After providing consent, they completed a sociodemographic questionnaire with items about their professional background; place of work; gender identity; and research experience in alcohol, tobacco, and behavioral science. They were then redirected to the first-round survey described previously.

After the first round, the research team sent the initial honorarium, analyzed the data, and created a summary of the survey responses that included data from the ordinal scale and the open-ended text fields. The team shared this summary with each participant when sending the second questionnaire, in which participants rerated the items and responded to existing comments if desired. The second-round survey included comments and points raised by panelists for each BCT, providing participants with the rationale underlying other panelists’ responses in the first-round survey. After completing the second round, the team sent panelists a thank-you email alongside their remaining honorarium.

### Data Analysis

Survey responses were included in the analysis if participants completed at least 1 survey question. Free-text responses from each round were collated and analyzed qualitatively using a thematic approach [39]. Two researchers independently reviewed all comments to identify recurring patterns and concepts and then met to discuss their findings. Through discussion, themes were developed that captured the key considerations and rationale provided by panelists regarding each BCT. These comments offered valuable context and helped clarify the rationale behind panelists’ agreement or disagreement with the BCTs. Several panelists contributed written comments across BCTs. Selected comments are presented in the Results section to highlight key themes and areas of concern.

Following each round, responses were summarized using measures of central tendency (median), distribution (range), and the proportion of ratings for each score assigned to each BCT. Although no universally accepted threshold exists for defining consensus, prior studies commonly apply a benchmark of approximately 70% to 80% agreement among participants [40].

For this study, a consensus threshold was defined a priori as follows:

- A BCT was considered “appropriate” for inclusion in the patient portal if at least 70% (10/14) of panelists rated it as “agree” across all 6 APEASE criteria.
- A BCT was considered “inappropriate” for inclusion in the patient portal if fewer than 50% (6/14) of panelists rated it as “agree” on ≥1 APEASE criteria.
- Partial consensus was defined as 50% (7/14) to 69% (9/14) agreement on ≥1 APEASE criteria.

This consensus approach has been successfully used in prior evaluations [33,41]. A qualitative analysis of free-text comments was conducted using a thematic approach to identify key patterns and insights [39]. Items that reached consensus as appropriate were included in the round 2 questionnaire with the points raised from the previous round and were rerated on the effectiveness criterion only. Items that reached consensus as inappropriate were not included in the round 2 questionnaire. For items that achieved partial consensus, the research team reviewed accompanying free-text comments and, based on this feedback, proposed revisions to improve their clarity and framing. These revised items constituted the round 2 questionnaire, along with a summary of points raised by panelists for each BCT that provided the rationale of other panelists when they answered the first survey.

### Ethical Considerations

This study was approved by the Centre for Addiction and Mental Health’s Quality Project Ethics Review (QPER-100). All participants were provided with a study briefing note and a digital consent form prior to participation. Informed consent was obtained electronically before completing the survey. To recognize the substantial time and expertise contributed by senior experts, panelists received a CAD $300 (CAD $1=US $0.71 as of Jun 18, 2026) honorarium. This was distributed in 2 installments: CAD $100 upon completion of round 1 and CAD $200 following completion of round 2. Depending on the participant’s preference, compensation was provided via an electronic gift card or electronic funds transfer.

## Results

### Expert Panel

Of the 48 experts invited to participate in the study, 29% (n=14) agreed to participate. Twelve (86%) experts completed the first round of the Delphi questionnaire, and 100% (n=14) of panelists participated in the second round. All 14 experts completed the sociodemographic survey. Overall, 14% (n=2) experts (panelists 6 and 11) did not complete the first round but participated in the second round, accounting for the discrepancy in participation between rounds. Invited experts who did not participate represented similar areas of expertise, including behavior change, tobacco and alcohol treatment, and digital interventions for substance use. All panelists were researchers with expertise in substance use, behavior change, and digital interventions. On average, participants reported 22.1 (SD 10) years of research experience related to alcohol, 17.0 (SD 11.2) years related to tobacco, and 23.2 (SD 10.2) years related to behavioral science. Sociodemographic characteristics of the panel are presented in Table 1.

**Table 1:** Sociodemographic characteristics (n=14).

Characteristic			Value
Total panelists, n (%)			14 (100)
Completed first survey, n (%)			12 (86)
Completed second survey, n (%)			14 (100)
**Gender, n (%)**			
	Men	8 (57)	
	Women	5 (36)	
	Not defined	1 (7)	
**Place of work, n (%)**			
	United States	8 (57)	
	United Kingdom	3 (21)	
	Canada	2 (14)	
	Not defined	1 (7)	
**Research experience (years), mean (SD)**			
	Alcohol	22.1 (10)	
	Tobacco	17 (11.2)	
	Behavioral science	23.2 (10.2)	

### First Round

Out of the 20 BCTs included in the first round, the panel reached consensus that 4 BCTs were appropriate for inclusion in a direct-to-patient digital intervention: *goal setting*, *action planning (individualized change plan)*, *action planning (reduction strategies)*, and *feedback on behavior*.

These techniques were considered practical, motivational, and feasible to implement digitally without real-time human support. However, 7 panelists offered several considerations regarding the application of these techniques. For example, in relation to goal setting, similar to comments made by other panelists, 1 panelist noted the following:

For the alcohol goal, it would be important to advise heavier drinking individuals to consult with a medical care provider before having an abstinence goal (to avoid dangerous withdrawal syndrome).

Panelist 8

Overall, 3 BCTs reached consensus as inappropriate for inclusion in a direct-to-patient digital intervention: *social support*, *social comparison*, and *comparative imagining of future outcomes*. Although 7 panelists recognized their potential value in the free-text comments, they raised concerns about their feasibility, the need for tailoring, or the cognitive burden associated with their inclusion in a BI. For example, for comparative imagining of future outcomes, 1 panelist commented the following:

I think this would not be valuable for the majority of people in the proposed context.

Panelist 4

These BCTs were also among those with the lowest equity ratings (details in Multimedia Appendix 2).

Partial consensus was achieved for 13 BCTs: *problem solving (identify barriers)*, *problem solving (craving strategies)*, *problem solving (identify situations)*, *information about health consequences*, *information about antecedents*, *information about antecedents (link between alcohol and smoking)*, *pros and cons*, *self-monitoring*, *behavioral substitution*, *information about social and environmental consequences*, *behavioral practice or rehearsal*, *credible source*, and *nonspecific reward.*

Panelists also emphasized that these techniques could inadvertently exacerbate inequities if delivered without appropriate tailoring, particularly in cases where delivery depends on self-guided engagement. With respect to problem solving (identify barriers), 1 participant cautioned as follows:

I think problem solving can be tricky in an online portal without someone helping to deliver the intervention and go through the different options.

Panelist 5

Two general key considerations were identified in the panelists’ free-text responses. First, panelists emphasized the importance of including only BCTs that could be implemented without clinician oversight, highlighting the need for feasibility in the context of a 1-time, self-guided digital intervention. Second, panelists stressed the importance of prioritization, raising concerns about the cognitive and logistical burden associated with delivering a large number of components simultaneously. One participant commented on problem solving (craving strategies):

This is a lot to cover in a portal. Perhaps consider the most effective and acceptable of these for most individuals.

Panelist 14

A summary of the round 1 results is presented in Table 2.

**Table 2:** First round consensus.

Consensus status and BCTs^a^		Domain (BCTTv1)	Panelist rationale or considerations
**Appropriate**			
	Goal setting (BCT 1.1; BCIO^b^:007002)	Goals and planning	“Participants will likely have goals to reduce alcohol but to stop smoking. Important to record those responses and to consider them in your analyses.”
	Action planning (individualized change plan; BCT 1.4; BCIO:007010)	Goals and planning	“Reduction in alcohol use should target hazardous drinking and reducing WHO risk drinking levels.”
	Action planning (reduction strategies; BCT 1.4; BCIO:007010)	Goals and planning	“Nice practical tips. Must be placed in context of high motivation to make potentially difficult life changes.”
	Feedback on behavior (BCT 2.2; *BCIO:007023)*	Feedback and monitoring	“There is a decent evidence base for social norms for alcohol use and other motivated behaviours. I think the norm messages need to be carefully constructed to avoid any unintended consequences (boomerang effects)”
**Partial**			
	Problem solving (identify barriers; BCT 1.2; BCIO:007008)	Goals and planning	“I think problem solving can be tricky in an online portal without someone helping to deliver the intervention and go through the different options.”
	Problem solving (craving strategies; BCT 1.2; BCIO:007008)	Goals and planning	Concern with a providing a lot of information in a one-time digital intervention expressed: “This is a lot to cover in a portal. Perhaps consider the most effective and acceptable of these for most individuals”
	Problem solving (identify situations; BCT 1.2; BCIO:007008)	Goals and planning	Concern with a one-time digital intervention expressed: “There needs to be some kind of role playing involved. You need to show videos and get the patient to do the skills, which are often challenging. You MUST cut off future offers for drinks/substances.”
	Information about health consequences (BCT 5.1; BCIO:007063)	Natural consequences	“Providing information on health consequences is often necessary but not sufficient for a change in behaviour, but given the rates of physical and mental health conditions in this group, I think it would be important to include.”
	Information about antecedents (BCT 4.2; BCIO:007052)	Shaping knowledge	“Identifying triggers is important but this may be challenging to accomplish using online portal approach”
	Information about antecedents (link between alcohol and smoking; BCT 4.2; BCIO:007052)	Shaping knowledge	“We do this clinically but I'm not sure whether there's actual research showing this to be effective”
	Pros and cons (BCT 9.2; BCIO:007069)	Comparison of outcomes	“I think this BCT can be improved if specific to the individual using the portal.”
	Self-monitoring (BCT 2.3; BCIO:007024)	Feedback and monitoring	“Thought to be effective but rarely seen reports of participants complying. Can be a bit complex to program unless you are just recommending but not providing a tracking portal.”
	Behavioral substitution (BCT 8.2; BCIO:007095)	Repetition and substitution	“For most people quitting smoking, it is finding alternatives to smoking that are challenging. I rarely have seen that in terms of people changing their drinking in the short term in order to boost smoking cessation success. Thus, this would be a low priority.”
	Information about social and environmental consequences (BCT 5.3; BCIO:007064)	Natural consequences	“I suspect people are already aware of these consequences so probably not the best use of the time available”
	Behavioral practice rehearsal (BCT 8.1; BCIO:007094)	Repetition and substitution	“There usually would not be time to do this before someone is actually making a quit smoking attempt. Furthermore, most people do not struggle with avoiding drinking for a short period. Therefore, this would be low priority.”
	Credible source (BCT 9.1; BCIO:007075)	Comparison of outcomes	“Not sure that such bibliotherapy will be helpful on its own but tying in to reducing hazardous drinking probably beneficial.”
	Nonspecific reward (BCT 10.3; BCIO:007252)	Reward and threat	“Really think about your target audience and the context you are designing the intervention for. Participants will be people primarily their from smoking cessation, most will not have severe alcohol consumption, and you do not have a lot of time.”
**Inappropriate**			
	Social support (BCT 3.1; BCIO:007028)	Social support	“Depends a lot on the social circle and the social care... I think this has the biggest impact on a BCT being equitable”
	Social comparison (BCT 6.2; BCIO:007073)	Comparison of behavior	“This would be relevant to very few people.”
	Comparative imagining of future outcomes (BCT 9.3; BCIO:007070)	Comparison of outcomes	“I think this would not be valuable for the majority of people in the proposed context.”

^a^BCT: behavior change technique.

^b^BCIO: Behaviour Change Intervention Ontology.

### Second Round

Out of the 14 BCTs included in the second round, 2 BCTs, *reattribution and pros and cons*, reached consensus as appropriate. Reattribution in this digital health context is defined as providing knowledge about what constitutes a standard drink and the safe drinking guidelines established by the World Health Organization to prompt users to reevaluate whether their drinking is “normal” or low-risk, thereby shifting their self-perception and perceived control over their behavior. This distinguishes it from purely informational techniques, which transfer knowledge without necessarily challenging causal beliefs or self-attribution, and from feedback-based techniques, which reflect observed behavior back to the user*.* Similar to comments shared by other panelists, 1 panelist pointed out how these techniques were consistent with guideline recommendations:

Providing such information is consistent with recommendations from NIAAA. It will be helpful to have links on your site for participants to connect with such outside information.

Panelist 12

It may not be necessary to go into the cons of reducing drinking. Simply checking off from a list of potential gains/benefits is likely sufficient.

Panelist 10

Five BCTS were deemed inappropriate: *problem solving (identify situations), behavioral substitution, information about social and environmental consequences, behavioral practice or rehearsal, and nonspecific reward*. Consistent with the concerns raised by other panelists, 1 panelist (panelist 10) commented that problem solving (identifying situations) “...sounds pretty involved and might be too intensive for the typical case.”

For behavioral substitution, 1 panelist (panelist 2) noted that suggested alternatives “would need some thought—also probably less equitable than others as these really depend on resources of the individual,” and recommended that “any suggestions offered should include options that are free or low cost.”

For nonspecific reward, 1 panelist (panelist 7) observed that “not all patients will have the same resources in terms of being able to reward themselves, potentially requiring money or time.”

Another panelist (panelist 5) cautioned that “rewards would need to be more powerful than drinking—might be difficult for some patients to conceptualise a type of reward that would work here.”

Another panelist commented regarding information about social and environmental consequences:

I don’t think this is the best use of resources/time in the portal.

Panelist 1

In total, 7 BCTs reached partial consensus: *problem solving (identify barriers), problem solving (craving strategies), information about health consequences, information about antecedents, information about antecedents (link between alcohol and smoking), self-monitoring, and credible source.* Similar to what other panelists had commented, 1 participant cautioned and agreed with fellow panelists’ previous points for information about antecedents and problem solving (identify barriers):

I think this one is one that I’d have most issues with the safety. Identifying these triggers in an unsupervised environment could possibly be dangerous.

Panelist 5

I agree with the points raised by panellists for this—the point around a one-time digital intervention is a good one. I also think this is far from equitable.

Panelist 5

Panelists highlighted that certain BCTs will only be effective if they are continued over time. As 1 participant mentioned regarding problem solving (craving strategies):

I think this is probably a lot for a one-time digital intervention. I don’t think it is an ineffective BCT, but I think it is something that would need continued engagement.

Panelist 5

Another point panelists emphasized was the importance of providing health information to participants. One participant commented the following:

It is important to provide at least minimal information on health risks when you have that opportunity.

Panelist 10

**Figure 1:**
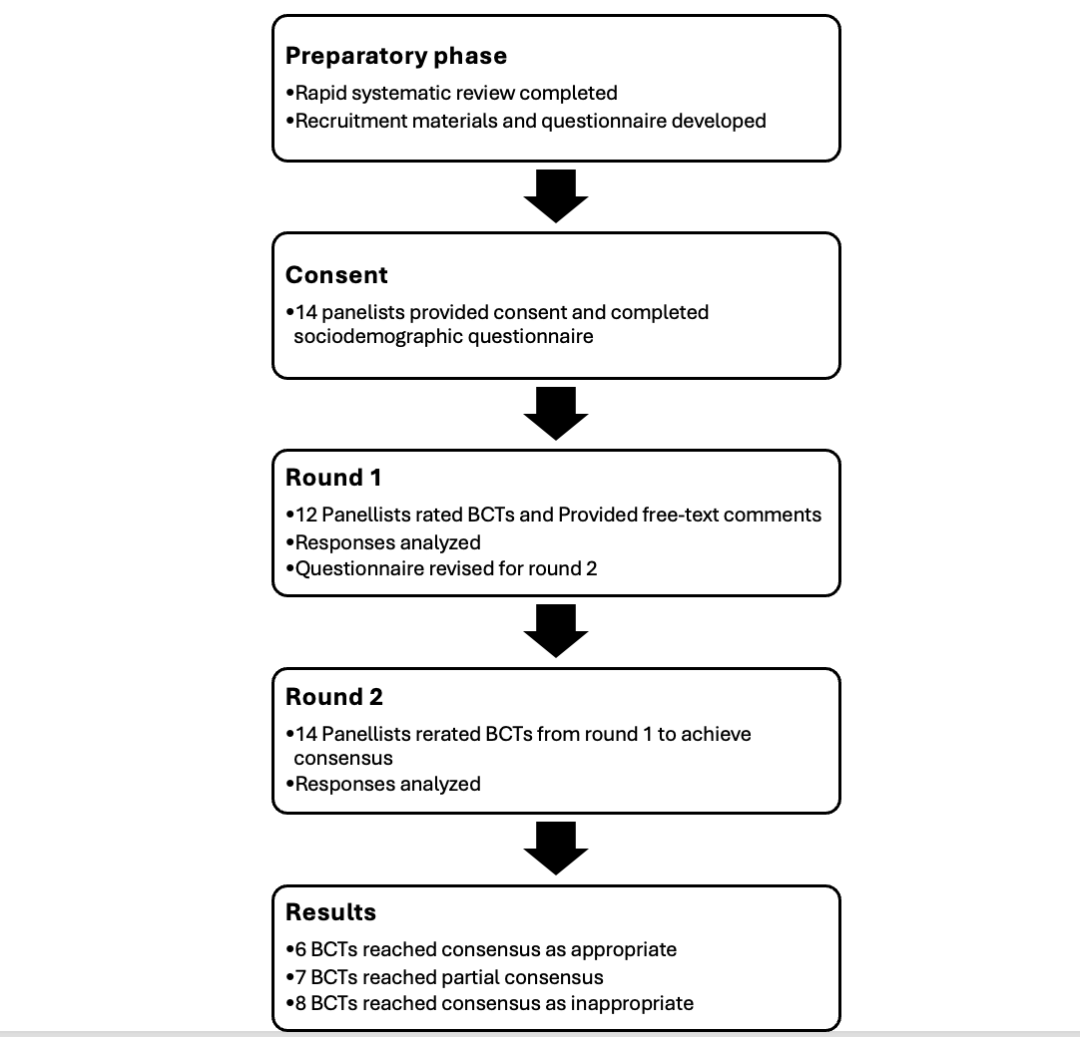
Delphi study process flowchart. BCT: behavior change technique.

The consensus ratings for the BCTs from the second survey are shown in Table 3, and the medians and consensus ratings for the BCTs across both rounds are shown in Multimedia Appendix 2. In total, based on the 2 rounds, 6 BCTs reached consensus as appropriate for inclusion in a patient portal targeting individuals with concurrent tobacco and alcohol use. An overview of the Delphi study process is shown in Figure 1.

**Table 3:** Second round consensus.

Consensus status and BCTs^a^		Domain (BCTTv1)	Panelist rationale or quote
**Appropriate**			
	Re-attribution (BCT 4.3; *BCIO*^b^:*007053*)	Shaping knowledge	“Providing such information is consistent with recommendations from NIAAA. It will be helpful to have links on your sit for participants to connect with such outside information.”
	Pros and cons (BCT 9.2; BCIO:007069)	Comparison of outcomes	“It may not be necessary to go into cons of reducing drinking. Simply checking off from a list of potential gains/benefits is likely sufficient.”
**Partial**			
	Problem solving (identify barriers; BCT 1.2; BCIO:007008)	Goals and planning	“I agree with the “points raised by panelists” section above. I struggle to understand how such problem solving will be implemented given this platform.”
	Problem solving (craving strategies; BCT 1.2; BCIO:007008)	Goals and planning	“I think this is probably a lot for a one-time digital intervention. I don't think it is an ineffective BCT, but I think it is something that would need continued engagement.”
	Information about health consequences (BCT 5.1; BCIO:007063)	Natural consequences	“It is important to provide at least minimal information on health risks when you have that opportunity.”
	Information about antecedents (BCT 4.2; BCIO:007052)	Shaping knowledge	“I think this one is one that I'd have most issues with the safety. Identifying these triggers in an unsupervised environment could possibly be dangerous.”
	Information about antecedents (link between alcohol and smoking; BCT 4.2; BCIO:007052)	Shaping knowledge	“I don't have any specific comments on this - largely because I'm not sure on the effectiveness in this context.”
	Self-monitoring (BCT 2.3; BCIO:007024)	Feedback and monitoring	“It would be hard to employ this in the portal unless it connected people to a text messaging program, which might be more than in practical to manage.”
	Credible source (BCT 9.1; BCIO:007075)	Comparison of outcomes	“I agree this is low burden. However, do people always listen to credible sources? Also, there will almost certainly be socio-demographic differences in this.”
**Inappropriate**			
	Problem solving (identify situations; BCT 1.2; BCIO:007008)	Goals and planning	“This sounds pretty involved and might be too intensive for the typical case.”
	Behavioral substitution (BCT 8.2; BCIO:007095)	Repetition and substitution	“I think this can be folded into strategies for limiting drinking. I don't know that going over substitutes in depth is needed other than, for example, suggesting alternating drinks with non-alcohol drinks or planning to consume only non-alcohol beverages at a given event.”
	Information about social and environmental consequences (BCT 5.3; BCIO:007064)	Natural consequences	“I don't think this is the best use of resources/time in the portal”
	Behavioral practice rehearsal (BCT 8.1; BCIO:007094)	Repetition and substitution	“I'm not sure that this strategy has been proven to be effective.”
	Nonspecific reward (BCT 10.3; BCIO:007252)	Reward and threat	“Not all patients will have the same resources in terms of being able to reward themselves (potentially requiring money or time).”

^a^BCT: behavior change technique.

^b^BCIO: Behaviour Change Intervention Ontology.

## Discussion

### Key Findings

This modified Delphi study aimed to identify BCTs that experts deemed appropriate for use in a 1-time, direct-to-patient digital intervention targeting dual use of alcohol and tobacco, with a specific focus on middle-aged adults with low income and education levels, those with a high prevalence of mental and physical health conditions, and those living in rural areas. Given these characteristics, the intervention must be simple, self-directed, and scalable, with minimal reliance on human facilitation or repeated engagement.

After 2 rounds, 6 BCTs achieved consensus as appropriate. Of these, 3 BCTs—goal setting (BCT 1.1; BCIO:007002) and the 2 variants of action planning (BCT 1.4; BCIO:007010)—fall within the Goals and Planning domain of the Behaviour Change Technique Taxonomy (version 1) [30]. The remaining techniques are distributed across other domains: Feedback and Monitoring (feedback on behavior: BCT 2.2; BCIO:007023), Shaping Knowledge (reattribution: BCT 4.3; BCIO:007053), and Comparison of Outcomes (pros and cons: BCT 9.2; BCIO:007069). This pattern reflects a preference for intervention strategies that emphasize goal-directed behavior, self-regulation, and intentional planning. These strategies enable individuals to translate intentions into action by setting specific goals, making concrete plans, and monitoring progress, which is particularly relevant in a direct-to-patient digital context where clinician involvement is minimal at the point of delivery, although patients may later have an opportunity to discuss their responses and plans with a health care professionals during follow-up care.

These findings are consistent with existing literature on digital behavior change interventions for substance use [42,43]. Goal setting and action planning are foundational self-regulation strategies and are among the most commonly used and effective BCTs for both smoking and alcohol reduction [44,45]. For example, in digital alcohol reduction interventions such as the Drink Less app [46], which includes modules for goal setting, self-monitoring, and pros and cons evaluation, users who engaged with goal-oriented content demonstrated more sustained reductions in alcohol use [47]. Although Drink Less involves repeated user engagement, the use of similar BCTs in a 1-time intervention may still serve as a low-touch prompt for behavior change, especially when grounded in motivational content and presented in a user-centered format. Digital interventions for smoking cessation, such as the Smoke Free app, also use self-monitoring, personalized feedback, and goal setting BCTs and have shown promise in promoting quit attempts and sustained abstinence [48]. Similarly, in a metaregression of physical activity and healthy eating interventions [49], the use of action planning and goal setting was significantly associated with greater behavior change. Although these findings were derived from a different behavioral domain (physical activity and healthy eating), they reinforce the theoretical value of these strategies across contexts. Reattribution and pros and cons target mechanisms such as causal attribution (BCIO:006032) and self-efficacy beliefs related to behavior (BCIO:006154). Although less frequently implemented in digital interventions, their selection reflects expert recognition of their motivational potential for the intended population.

The BCTs *reattribution* and *pros and cons* also achieved consensus for inclusion. These techniques align with theoretical frameworks such as the health belief model and the transtheoretical model, which emphasize *cognitive restructuring* and *decisional balance* as precursors to behavior change. Reattribution helps individuals reinterpret the causes of their behavior, potentially reducing fatalism and increasing perceived control, which are important constructs in substance use contexts [50,51]. Similarly, pros and cons is a well-established decisional balance technique that helps users reflect on the positive and negative consequences of behavior change. It engages mechanisms of evaluative belief (BCIO:006148) and readiness to change [22]. These strategies have been used successfully in apps such as Drink Less and the UK National Health Service Smokefree app, which includes a decisional balance tool for evaluating the advantages of quitting smoking [52,53]. Although reattribution is less commonly used in interventions [54], it was considered appropriate in this case, given the target population’s need for motivational support and increased self-efficacy. Research suggests that individuals who use both alcohol and tobacco are more likely to experience motivational barriers and fatalistic beliefs, which can make behavior change more difficult [55]. This highlights the importance of addressing these psychological factors through BCTs such as reattribution.

The expert panel also reached consensus on 8 BCTs as inappropriate in this context: social support (BCT 3.1; BCIO:007028), social comparison (BCT 6.2; BCIO:007073), comparative imagining of future outcomes (BCT 9.3; BCIO:007070), problem solving (identify situations: BCT 1.2; BCIO:007008), behavioral substitution (BCT 8.2; BCIO:007095), information about social and environmental consequences (BCT 5.3; BCIO:007064), behavioral practice rehearsal (BCT 8.1; BCIO:007094), and nonspecific reward (BCT 10.3; BCIO:007252). Experts expressed that these BCTs were less feasible due to their reliance on social interaction, personalization, or repeated engagement, which are incompatible with a 1-time, automated intervention. It is important to note that these techniques may still be valuable in other formats, such as clinician-delivered interventions or multisession digital programs, and their exclusion here reflects contextual limitations rather than their intrinsic effectiveness. However, given the constraints of a 1-time digital intervention, especially for populations with limited time, goal setting, action planning, and feedback on behavior emerged as the highest priority BCTs. These techniques were consistently rated as both effective and feasible, offering the greatest potential for impact within a brief, self-guided format. If intervention time or space is restricted, prioritizing these core self-regulation strategies is recommended.

Equity considerations appeared to further differentiate BCTs that failed to reach consensus from those that were ultimately included. Specifically, BCTs that did not reach consensus were more frequently characterized by panelists as requiring greater cognitive effort, sustained engagement, social resources, or material supports—factors that may disproportionately disadvantage individuals with lower socioeconomic status or limited digital access. This pattern is consistent with existing literature demonstrating that digital behavior change interventions can inadvertently widen health inequities when intervention components are more accessible or actionable for individuals with greater resources, higher health literacy, or stronger digital skills [56].

### Strengths and Limitations

A key strength of this study is the diversity of the expert panel in terms of disciplinary background (behavioral science and substance use) and geographic representation, including participants from multiple countries. This broad perspective enhances the relevance of the findings to global digital health contexts and supports the design of 1-time digital interventions that address both substances. At the same time, the panel was composed exclusively of researchers. Although research expertise provides a valuable and rigorous foundation for early-stage intervention design, the absence of clinician and patient perspectives may have skewed BCT selection toward techniques that are theoretically robust but not necessarily optimal in practice. Given that the intervention is intended for direct use by patients enrolled in the STOP program, many of whom have low income and education levels, a high burden of mental and physical health conditions, and limited experience with digital health tools, patient perspectives on engagement, comprehensibility, and feasibility will be essential for further refinement.

The use of the APEASE criteria similarly reflects both a strength and a limitation. By requiring panelists to systematically evaluate affordability, practicability, effectiveness, acceptability, safety, and equity, the framework ensured that BCT selection was not driven by effectiveness evidence alone, representing a meaningful departure from conventional approaches to intervention design. A limitation of this study is that the findings are based on expert consensus rather than empirical evaluation of intervention effectiveness. Although the Delphi method is well suited to identifying BCTs perceived as appropriate and feasible, it does not provide evidence of their actual impact on behavior change outcomes. Therefore, the BCTs identified in this study should be interpreted as promising candidates for inclusion rather than as components proven to be effective in practice. The explicit inclusion of equity is particularly notable, as it highlights concerns about differential accessibility that are often absent from BCT selection processes. However, prior research has shown that digital health interventions can inadvertently contribute to intervention-generated inequalities when access, engagement, or benefit are patterned by socioeconomic status, digital literacy, or resource availability [57], and the equity assessments in this study were based on expert judgment rather than direct evidence from underserved groups. As such, the equity-related findings should be interpreted as hypothesis generating rather than definitive and require validation through empirical and participatory research. A qualitative study is planned to explore patients’ and health care professionals’ perspectives on the intervention’s design, with findings intended to inform future iterations and ensure that expert consensus is translated into content that is genuinely accessible and acceptable to the intended population.

### Conclusions

This modified Delphi study identified a core set of BCTs (centered on goal setting, planning, feedback, and decisional balance) that experts judged to be appropriate for a 1-time, direct-to-patient digital intervention targeting dual alcohol and tobacco use. These findings provide preliminary guidance for the development of brief digital tools aimed at promoting self-regulation and behavior change in real-world, resource-constrained settings. Although expert consensus provides a useful foundation, future research should integrate patient perspectives and usability testing to ensure that these techniques resonate with intended users and support meaningful behavior change.

## Appendix

Multimedia Appendix 1 Delphi study briefing note.

Multimedia Appendix 2 Median and consensus percentages of panelists for behavior change techniques.

## Abbreviations

APEASE: acceptability, practicability, effectiveness, affordability, safety, and equity
BCIO: Behaviour Change Intervention Ontology
BCT: behavior change technique
BI: brief intervention
CREDES: Conducting and Reporting Delphi Studies
eSBI: electronic screening and brief intervention
PAR: population attributable risk
REDCap: Research Electronic Data Capture
STOP: Smoking Treatment for Ontario Patients
