# Supplementary material for: Identifying Behavior Change Techniques for Digital Interventions Addressing Alcohol and Tobacco Co-Use: Findings From a Delphi Consensus Study
Source: JMIR Form Res. 2026 Jul 3;10:e88996. doi: 10.2196/88996 (PMC13379691; doi:10.2196/88996)
Supplement: Multimedia Appendix 1 [file formative_v10i1e88996_app1.pdf]

Delphi Panel Overview

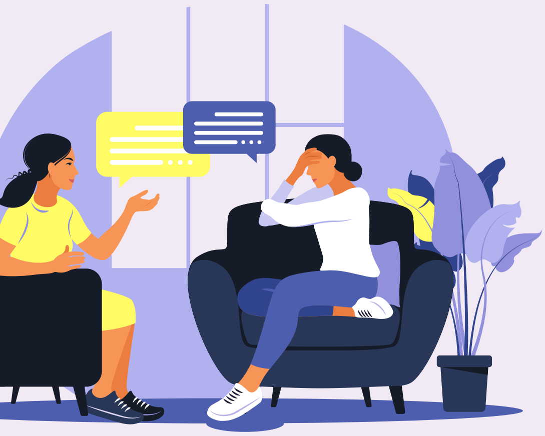

What is the Smoking Treatment for Ontario Patients (STOP) Program?

The STOP Program, funded by the Ontario Ministry of Health, is a province-wide initiative delivering smoking cessation treatment (up to 26 weeks of Nicotine Replacement Therapy) and counselling support to people who want to reduce/quit their tobacco use. These supports are available free of charge, through partnerships with community health care organizations. Importantly, STOP has been able to reach and successfully be implemented within vulnerable communities that face barriers in accessing care.

STOP Program patients' demographics:

- Average age of 53 years (half patients between 41 and 64 years)
- 24% did not complete high school
- 61% have household incomes of $40,000 or less
- People from rural areas are overrepresented
- 10% are permanently disabled
- 55% report lifetime diagnoses of anxiety or depression
- 46% report physical health conditions
- 21% report substance use problems (other than tobacco and caffeine)

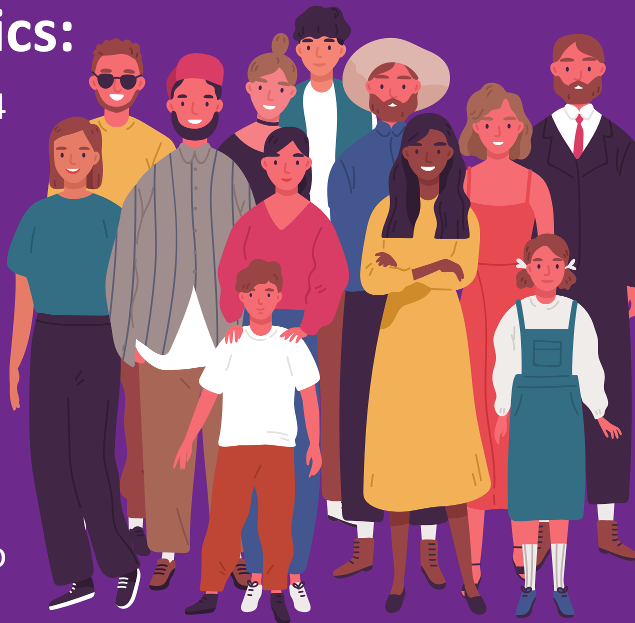

What will be asked of you in the Delphi Panel?

Our rapid review identified a number behavior change techniques (BCTs) for addressing dual alcohol and tobacco use. We are asking you to help us **evaluate** their inclusion in the STOP Program patient portal. You will be asked to complete a **brief demographic questionnaire** and then indicate your **agreement or disagreement** with each proposed intervention strategy based on the **APEASE** criteria (acceptability, practicability, effectiveness, affordability, safety, and equity) in **two rounds** to achieve consensus.

Compensation will be provided for participation

CIHR IRSC Canadian Institutes of Health Research Instituts de recherche en santé du Canada camh INTREPID Lab

Delphi Panel Overview

Behavior Change Techniques (BCTs) identified from the rapid review

BCTs	Definiton
Goal Setting	Allow/support patients to set goals to reduce or quit alcohol consumption by setting reduction goal and/or quit date
Action planning	Support patients by helping them develop an individualized change plan
Problem Solving	Help them identify barriers to making change
Information on health consequences	Provide information about the harmful effects of hazardous alcohol use and the health benefits of reducing or stopping alcohol consumption
Feedback on behaviour	Provide personalized feedback to patients about their level and patterns of alcohol consumption
Information about antecedents	Provide information about behavioral antecedents to alcohol consumption
Pros and cons	Encourage patients to explore the pros and cons of reducing or stopping alcohol consumption
Self-monitoring	Encourage patients to monitor their alcohol consumption
Social support	Encourage patients to arrange the help of their social circle in reducing or stopping alcohol consumption

BCTs	Definiton
Social comparison	Provide information about others who have quit drinking to help patients appreciate that they can find a solution to their problems
Behaviour substitution	Encourage patients to adopt a substitute/alternative activity for alcohol use
Information about social and environmental consequences	Provide information about social and environmental consequences of harmful alcohol consumption
Behavioural practice rehearsal	Encourage participants to practice their skills to reduce or stop alcohol use
Credible source	Mention credible sources of information provided in the portal
Comparative imagining of future outcomes	Encourage patients to imagine life without harmful alcohol use
Non-specific reward	Encourage patients to reward themselves for progress toward their alcohol consumption reduction/cessation goals

If you are interested in our study or if you have any questions, please feel free to contact

idat.study@camh.ca

Funding for this project is provided by the CIHR Institute of Cancer Research (ICR)
CIHR Grant # DCP-192068

CIHR IRSC Canadian Institutes of Health Research Instituts de recherche en santé du Canada

camh INTREPID Lab
