# Supplementary material for: Identifying Behavior Change Techniques for Digital Interventions Addressing Alcohol and Tobacco Co-Use: Findings From a Delphi Consensus Study
Source: JMIR Form Res. 2026 Jul 3;10:e88996. doi: 10.2196/88996 (PMC13379691; doi:10.2196/88996)
Supplement: Multimedia Appendix 2 [file formative_v10i1e88996_app2.docx]

**Table S1. Median & Consensus Percentage of Panelists for BCTs**

| **BCTs** | **Round 1- Median** | **Round 1 -** % of panelists who agreed or strongly agreed that the BCT should be included | **Round 2- Median** | **Round 2-** % of panelists who agreed or strongly agreed that the BCT should be included |
| --- | --- | --- | --- | --- |
| **Goal setting**  **(*Only Assessed Effectiveness in Round 2)** | | | | |
| Acceptability | 1.5 ( Strongly agree) | 92 |  |  |
| Practicability | 1.5 (Strongly agree) | 92 |  |  |
| Effectiveness | 2 (Agree) | 83 | 1 (Strongly agree) | 100 |
| Affordability | 1 (Strongly agree) | 100 |  |  |
| Safety | 1.5 (Strongly agree) | 92 |  |  |
| Equitability | 1 (Strongly agree) | 92 |  |  |
| **Action planning (Individualized change plan) (*Only Assessed Effectiveness in Round 2)** | | | | |
| **BCTs** | **Round 1- Median** | **Round 1 -** % of panelists who agreed or strongly agreed that the BCT should be included | **Round 2- Median** | **Round 2-** % of panelists who agreed or strongly agreed that the BCT should be included |
| Acceptability | 2 (Agree) | 100 |  |  |
| Practicability | 2 (Agree) | 75 |  |  |
| Effectiveness | 2 (Agree) | 100 | 1 (Strongly agree) | 100 |
| Affordability | 1.5 (Strongly agree) | 83 |  |  |
| Safety | 1 (Strongly agree) | 92 |  |  |
| Equitability | 2 (Agree) | 75 |  |  |
| **Action Planning (Reduction strategies) (*Only Assessed Effectiveness in Round 2)** | | | | |
| **BCTs** | **Round 1- Median** | **Round 1 -** % of panelists who agreed or strongly agreed that the BCT should be included | **Round 2- Median** | **Round 2-** % of panelists who agreed or strongly agreed that the BCT should be included |
| Acceptability | 2 (Agree) | 83 |  |  |
| Practicability | 1 (Strongly agree) | 92 |  |  |
| Effectiveness | 1 (Strongly agree) | 75 | 1.5 (Strongly agree) | 71 |
| Affordability | 1 (Strongly agree) | 91 |  |  |
| Safety | 2 (Agree) | 75 |  |  |
| Equitability | 2 (Agree) | 92 |  |  |
| **Re-attribution (*Included in Round 2)** | | | | |
| **BCTs** | **Round 1- Median** | **Round 1 -** % of panelists who agreed or strongly agreed that the BCT should be included | **Round 2- Median** | **Round 2-** % of panelists who agreed or strongly agreed that the BCT should be included |
| Acceptability |  |  | 1 (Strongly agree) | 93 |
| Practicability |  |  | 2 (Agree) | 100 |
| Effectiveness |  |  | 2 (Agree) | 71 |
| Affordability |  |  | 1 (Strongly agree) | 93 |
| Safety |  |  | 1 (Strongly agree) | 86 |
| Equitability |  |  | 2 (Agree) | 71 |
| **Problem solving (Identify barriers)** | | | | |
| **BCTs** | **Round 1- Median** | **Round 1 -** % of panelists who agreed or strongly agreed that the BCT should be included | **Round 2- Median** | **Round 2-** % of panelists who agreed or strongly agreed that the BCT should be included |
| Acceptability | 1 (Strongly agree) | 83 | 2 (Agree) | 86 |
| Practicability | 2 (Agree) | 67 | 2.5 (Neutral) | 50 |
| Effectiveness | 1.5 (Strongly agree) | 83 | 2 (Agree) | 64 |
| Affordability | 1.5 (Strongly agree) | 83 | 2 (Agree) | 64 |
| Safety | 1.5 (Strongly agree) | 83 | 2 (Agree) | 86 |
| Equitability | 2 (Agree) | 75 | 2 (Agree) | 57 |
| **Problem solving (Craving strategies)** | | | | |
| **BCTs** | **Round 1- Median** | **Round 1 -** % of panelists who agreed or strongly agreed that the BCT should be included | **Round 2- Median** | **Round 2-** % of panelists who agreed or strongly agreed that the BCT should be included |
| Acceptability | 2 (Agree) | 92 | 2 (Agree) | 86 |
| Practicability | 2 (Agree) | 67 | 2.5 (Neutral) | 50 |
| Effectiveness | 2 (Agree) | 75 | 2 (Agree) | 71 |
| Affordability | 1.5 (Strongly agree) | 83 | 2 (Agree) | 71 |
| Safety | 2 (Agree) | 83 | 2 (Agree) | 93 |
| Equitability | 2 (Agree) | 83 | 2 (Agree) | 79 |
| **Problem solving (Identify situations)** | | | | |
| **BCTs** | **Round 1- Median** | **Round 1 -** % of panelists who agreed or strongly agreed that the BCT should be included | **Round 2- Median** | **Round 2-** % of panelists who agreed or strongly agreed that the BCT should be included |
| Acceptability | 2 (Agree) | 83 | 2 (Agree) | 71 |
| Practicability | 2 (Agree) | 83 | 3 (Neutral) | 36 |
| Effectiveness | 1 (Strongly agree) | 67 | 3 (Neutral) | 43 |
| Affordability | 1.5 (Strongly agree) | 83 | 2 (Agree) | 71 |
| Safety | 1.5 (Strongly agree) | 92 | 2 (Agree) | 93 |
| Equitability | 2 (Agree) | 75 | 2 (Agree) | 71 |
| **Information about health consequences** | | | | |
| **BCTs** | **Round 1- Median** | **Round 1 -** % of panelists who agreed or strongly agreed that the BCT should be included | **Round 2- Median** | **Round 2-** % of panelists who agreed or strongly agreed that the BCT should be included |
| Acceptability | 1 (Strongly agree) | 100 | 2 (Agree) | 100 |
| Practicability | 1 (Strongly agree) | 100 | 1 (Strongly agree) | 93 |
| Effectiveness | 2 (Agree) | 58 | 2 (Agree) | 57 |
| Affordability | 1 (Strongly agree) | 92 | 1 (Strongly agree) | 100 |
| Safety | 1 (Strongly agree) | 83 | 1 (Strongly agree) | 93 |
| Equitability | 1 (Strongly agree) | 83 | 1.5 (Strongly agree) | 79 |
| **Feedback on behaviour (*Only Assessed Effectiveness in Round 2)** | | | | |
| **BCTs** | **Round 1- Median** | **Round 1 -** % of panelists who agreed or strongly agreed that the BCT should be included | **Round 2- Median** | **Round 2-** % of panelists who agreed or strongly agreed that the BCT should be included |
| Acceptability | 1 (Strongly agree) | 100 |  |  |
| Practicability | 2 (Agree) | 92 |  |  |
| Effectiveness | 1.5 (Strongly agree) | 83 | 1 (Strongly agree) | 100 |
| Affordability | 1 (Strongly agree) | 83 |  |  |
| Safety | 1.5 (Strongly agree) | 83 |  |  |
| Equitability | 1.5 (Strongly agree) | 75 |  |  |
| **Information about antecedents** | | | | |
| **BCTs** | **Round 1- Median** | **Round 1 -** % of panelists who agreed or strongly agreed that the BCT should be included | **Round 2- Median** | **Round 2-** % of panelists who agreed or strongly agreed that the BCT should be included |
| Acceptability | 2 (Agree) | 83 | 2 (Agree) | 79 |
| Practicability | 2 (Agree) | 67 | 2.5 (Neutral) | 50 |
| Effectiveness | 2 (Agree) | 67 | 2 (Agree) | 71 |
| Affordability | 1 (Strongly agree) | 92 | 2 (Agree) | 64 |
| Safety | 1.5 (Strongly agree) | 75 | 2 (Agree) | 79 |
| Equitability | 2 (Agree) | 67 | 2 (Agree) | 64 |
| **Information about antecedents (link between alcohol and smoking)** | | | | |
| **BCTs** | **Round 1- Median** | **Round 1 -** % of panelists who agreed or strongly agreed that the BCT should be included | **Round 2- Median** | **Round 2-** % of panelists who agreed or strongly agreed that the BCT should be included |
| Acceptability | 2 (Agree) | 83 | 1 (Strongly agree) | 100 |
| Practicability | 2 (Agree) | 100 | 1 (Strongly agree) | 100 |
| Effectiveness | 2 (Agree) | 67 | 2 (Agree) | 64 |
| Affordability | 1 (Strongly agree) | 100 | 1 (Strongly agree) | 100 |
| Safety | 2 (Agree) | 92 | 1 (Strongly agree) | 100 |
| Equitability | 2 (Agree) | 75 | 1 (Strongly agree) | 93 |
| **Pros and cons** | | | | |
| **BCTs** | **Round 1- Median** | **Round 1 -** % of panelists who agreed or strongly agreed that the BCT should be included | **Round 2- Median** | **Round 2-** % of panelists who agreed or strongly agreed that the BCT should be included |
| Acceptability | 1.5 (Strongly agree) | 92 | 2 (Agree) | 86 |
| Practicability | 2 (Agree) | 75 | 2 (Agree) | 79 |
| Effectiveness | 2 (Agree) | 67 | 2 (Agree) | 79 |
| Affordability | 2 (Agree) | 75 | 1.5 (Strongly agree) | 86 |
| Safety | 1 (Strongly agree) | 100 | 1.5 (Strongly agree) | 86 |
| Equitability | 1.5 (Strongly agree) | 75 | 2 (Agree) | 79 |
| **Self-monitoring** | | | | |
| **BCTs** | **Round 1- Median** | **Round 1 -** % of panelists who agreed or strongly agreed that the BCT should be included | **Round 2- Median** | **Round 2-** % of panelists who agreed or strongly agreed that the BCT should be included |
| Acceptability | 1 (Strongly agree) | 83 | 2 (Agree) | 64 |
| Practicability | 2 (Agree) | 67 | 2.5 (Neutral) | 50 |
| Effectiveness | 2 (Agree) | 75 | 2 (Agree) | 64 |
| Affordability | 1.5 (Strongly agree) | 75 | 2 (Agree) | 79 |
| Safety | 1 (Strongly agree) | 92 | 1.5 (Strongly agree) | 100 |
| Equitability | 1 (Strongly agree) | 92 | 2 (Agree) | 100 |
| **Social support (*Removed in Round 2)** | | | | |
| **BCTs** | **Round 1- Median** | **Round 1 -** % of panelists who agreed or strongly agreed that the BCT should be included | **Round 2- Median** | **Round 2-** % of panelists who agreed or strongly agreed that the BCT should be included |
| Acceptability | 2.5 (Neutral) | 50 |  |  |
| Practicability | 3 (Neutral) | 25 |  |  |
| Effectiveness | 2 (Agree) | 58 |  |  |
| Affordability | 1.5 (Strongly agree) | 58 |  |  |
| Safety | 2.5 (Neutral) | 50 |  |  |
| Equitability | 2 (Agree) | 58 |  |  |
| **Social comparison (*Removed in Round 2)** | | | | |
| **BCTs** | **Round 1- Median** | **Round 1 -** % of panelists who agreed or strongly agreed that the BCT should be included | **Round 2- Median** | **Round 2-** % of panelists who agreed or strongly agreed that the BCT should be included |
| Acceptability | 2.5 (Neutral) | 50 |  |  |
| Practicability | 2.5 (Neutral) | 50 |  |  |
| Effectiveness | 3 (Neutral) | 8 |  |  |
| Affordability | 2.5 (Neutral) | 50 |  |  |
| Safety | 2 (Agree) | 75 |  |  |
| Equitability | 3 (Neutral) | 33 |  |  |
| **Behavioural substitution (*Removed in Round 2)** | | | | |
| **BCTs** | **Round 1- Median** | **Round 1 -** % of panelists who agreed or strongly agreed that the BCT should be included | **Round 2- Median** | **Round 2-** % of panelists who agreed or strongly agreed that the BCT should be included |
| Acceptability | 2 (Agree) | 92 | 2 (Agree) | 71 |
| Practicability | 2 (Agree) | 92 | 2 (Agree) | 64 |
| Effectiveness | 2 (Agree) | 67 | 2 (Agree) | 57 |
| Affordability | 2 (Agree) | 75 | 3 (Neutral) | 43 |
| Safety | 1.5 (Strongly agree) | 92 | 1 (Strongly agree) | 71 |
| Equitability | 2.5 (Neutral) | 50 | 2.5 (Neutral) | 50 |
| **Information about social and environmental consequences** | | | | |
| **BCTs** | **Round 1- Median** | **Round 1 -** % of panelists who agreed or strongly agreed that the BCT should be included | **Round 2- Median** | **Round 2-** % of panelists who agreed or strongly agreed that the BCT should be included |
| Acceptability | 2 (Agree) | 75 | 2.5 (Neutral) | 50 |
| Practicability | 2 (Agree) | 67 | 2 (Agree) | 71 |
| Effectiveness | 2.5 (Neutral) | 50 | 3 (Neutral) | 36 |
| Affordability | 2 (Agree) | 67 | 2 (Agree) | 71 |
| Safety | 2 (Agree) | 67 | 2 (Agree) | 86 |
| Equitability | 2 (Agree) | 67 | 2 (Agree) | 71 |
| **Behavioural practice rehearsal** | | | | |
| **BCTs** | **Round 1- Median** | **Round 1 -** % of panelists who agreed or strongly agreed that the BCT should be included | **Round 2- Median** | **Round 2-** % of panelists who agreed or strongly agreed that the BCT should be included |
| Acceptability | 2 (Agree) | 75 | 2 (Agree) | 57 |
| Practicability | 2 (Agree) | 67 | 3 (Neutral) | 43 |
| Effectiveness | 2 (Agree) | 67 | 2 (Agree) | 64 |
| Affordability | 2 (Agree) | 67 | 2 (Agree) | 71 |
| Safety | 1 (Strongly agree) | 92 | 2 (Agree) | 79 |
| Equitability | 2 (Agree) | 67 | 2 (Agree) | 64 |
| **Credible source** | | | | |
| **BCTs** | **Round 1- Median** | **Round 1 -** % of panelists who agreed or strongly agreed that the BCT should be included | **Round 2- Median** | **Round 2-** % of panelists who agreed or strongly agreed that the BCT should be included |
| Acceptability | 1 (Strongly agree) | 100 | 1 (Strongly agree) | 93 |
| Practicability | 1.5 (Strongly agree) | 83 | 1 (Strongly agree) | 93 |
| Effectiveness | 2.5 (Neutral) | 50 | 2 (Agree) | 64 |
| Affordability | 1.5 (Strongly agree) | 83 | 1 (Strongly agree) | 93 |
| Safety | 1 (Strongly agree) | 92 | 1 (Strongly agree) | 86 |
| Equitability | 2 (Agree) | 67 | 1.5 (Strongly agree) | 79 |
| **Comparative imagining of future outcomes (*Removed in Round 2)** | | | | |
| **BCTs** | **Round 1- Median** | **Round 1 -** % of panelists who agreed or strongly agreed that the BCT should be included | **Round 2- Median** | **Round 2-** % of panelists who agreed or strongly agreed that the BCT should be included |
| Acceptability | 2 (Agree) | 67 |  |  |
| Practicability | 2 (Agree) | 67 |  |  |
| Effectiveness | 3 (Neutral) | 42 |  |  |
| Affordability | 1.5 (Strongly agree) | 75 |  |  |
| Safety | 1 (Strongly agree) | 75 |  |  |
| Equitability | 2 (Agree) | 67 |  |  |
| **Non-specific reward** | | | | |
| **BCTs** | **Round 1- Median** | **Round 1 -** % of panelists who agreed or strongly agreed that the BCT should be included | **Round 2- Median** | **Round 2-** % of panelists who agreed or strongly agreed that the BCT should be included |
| Acceptability | 2 (Agree) | 100 | 2 (Agree) | 79 |
| Practicability | 2 (Agree) | 67 | 2 (Agree) | 57 |
| Effectiveness | 2 (Agree) | 58 | 2.5 (Neutral) | 50 |
| Affordability | 2 (Agree) | 58 | 3 (Neutral) | 43 |
| Safety | 1.5 (Strongly agree) | 83 | 2 (Agree) | 93 |
| Equitability | 2 (Agree) | 58 | 2.5 (Neutral) | 50 |
